# Development of a Method for Commercial Style Transfer of Historical Architectural Facades Based on Stable Diffusion Models

**DOI:** 10.3390/jimaging10070165

**Published:** 2024-07-11

**Authors:** Jiaxin Zhang, Yiying Huang, Zhixin Li, Yunqin Li, Zhilin Yu, Mingfei Li

**Affiliations:** 1 Architecture and Design College, Nanchang University, Nanchang 330031, China; jiaxin.arch@ncu.edu.cn (J.Z.); 6011120021@email.ncu.edu.cn (Y.H.); 6011121010@email.ncu.edu.cn (Z.L.); liyunqin@ncu.edu.cn (Y.L.); 2 Division of Sustainable Energy and Environmental Engineering, Graduate School of Engineering, Osaka University, Suita 565-0871, Japan; 3 School of Architecture, Southeast University, Nanjing 210096, China; 220220060@seu.edu.cn

**Keywords:** historical architectural facades, stable diffusion model, commercial style transfer, low-rank adaptation model

## Abstract

In the sphere of urban renewal of historic districts, preserving and innovatively reinterpreting traditional architectural styles remains a primary research focus. However, the modernization and adaptive reuse of traditional buildings often necessitate changes in their functionality. To cater to the demands of tourism in historic districts, many traditional residential buildings require conversion to commercial use, resulting in a mismatch between their external form and their internal function. This study explored an automated approach to transform traditional residences into commercially viable designs, offering an efficient and scalable solution for the modernization of historic architecture. We developed a methodology based on diffusion models, focusing on a dataset of nighttime shopfront facades. By training a low-rank adaptation (LoRA) model and integrating the ControlNet model, we enhanced the accuracy and stability of the generated images. The methodology’s performance was validated through qualitative and quantitative assessments, optimizing the batch size, repetition, and learning rate configurations. These evaluations confirmed the method’s effectiveness. Our findings significantly advance the modern commercial style transformation of historical architectural facades, providing a novel solution that maintains the aesthetic and functional integrity, thereby fostering breakthroughs in traditional design thinking and exploring new possibilities for the preservation and commercial adaptation of historical buildings.

## 1. Introduction

Historical architecture serves as a testament to history, encapsulating substantial cultural and artistic value, reflecting the historical context, societal customs, and aesthetic preferences of a region [1]. However, with rapid societal development and modernization, these structures face numerous challenges, including inadequate functionality for contemporary needs, structural safety concerns, and elevated maintenance costs [2]. Therefore, it is critical to transform the historical architecture to modern forms in order to satisfy the contemporary needs while addressing the safety and maintenance issues. To achieve this goal, prior work has focused on balancing the preservation of historical architecture with contemporary demands. For example, Bullen and Love [3] proposed the modernization of historical architecture not only to protect heritage but also to enhance the functionality, safety, and environmental sustainability of cultural districts. This approach facilitates the practical use of historical sites while ensuring their preservation.

With the rise of experiential tourism, traditional historical districts have become crucial locales for tourist experiences and serve as conduits for innovative business models. These cultural districts vividly display culture and history, enrich cultural content, and contribute to the preservation and transmission of culture [4]. They can also transform into centers of social and economic vitality [5], thereby accommodating modernization needs. Commercial activities significantly impact the renovation of traditional buildings, enhancing the vibrancy of historical districts. Historically, residential buildings within these districts primarily served as living spaces. However, the increasing demands of tourism and commerce have shifted their function from residential to commercial [6]. This transformation has led to a divergence between the buildings’ original residential purpose and their new commercial use. As such, the adaptation of traditional residential architecture for commercial purposes is not only significant but necessary, catering to the evolving needs of historical cultural districts. This process requires a delicate balance between preserving historical values and integrating modern commercial functionalities, presenting a complex challenge in urban cultural conservation [7]. This transformation underscores the dynamic interplay between maintaining historical integrity and meeting contemporary economic demands.

Building on the discussion of the commercial transformation of traditional residential architecture, it is essential to consider broader principles of historical architecture restoration. The Athens Charter, for instance, stresses that new constructions in proximity to historical districts should harmonize with the traditional aesthetic, preserving the historical period’s visual continuity [8]. This principle demands that architects adopt a macroscopic perspective, deeply understand and respect historical culture, and challenge their capability to manage buildings from different historical contexts and cultural districts [9]. In the field of architecture, while it is possible to categorize facade elements, precisely fitting elements of individual buildings or applying strict mathematical proportions remains a daunting task. This process is not only time-consuming and labor-intensive but also challenging to ensure the accuracy and objectivity of the assessments.

The complexity of China’s traditional architectural system, shaped by regional variations, historical transitions, and diverse design philosophies, poses significant challenges to the preservation and modernization of heritage buildings. One major challenge is the often insufficient understanding of traditional construction techniques among architects, which can lead to renovations that deviate significantly from traditional aesthetics, thus disrupting the cohesive appearance of architectural complexes [10]. In recent years, the urgent need for the preservation of historical architecture has necessitated optimizing resource allocation and conserving human efforts. This shift has emphasized the importance of freeing up human resources from labor-intensive tasks such as data organization, allowing more focus on core preservation activities and innovative design. Additionally, the commercial transformation of traditional architectural facades introduces further complexities. These transformations need to accommodate modern functional requirements while preserving and promoting historical and cultural values. The aesthetic form of the facade, playing a decisive role in defining the architectural character, must integrate style, proportion, and modern commercial needs without compromising the traditional integrity. Together, these challenges underscore the delicate balance required between modernization demands and traditional values, demanding a deep understanding of historical contexts and innovative approaches to both design and construction.

Navigating these architectural challenges necessitates not only a deep appreciation of historical aesthetics but also the adoption of innovative tools. With advancements in artificial intelligence, particularly through the development of generative adversarial networks (GANs) by Goodfellow et al. [11], and further innovations like CycleGAN by Zhu et al. [12], the architecture industry is witnessing a transformation in the design of historical districts. These AI models enable the creation of high-resolution images and the transformation of existing images to match the aesthetics of historical districts while preserving semantic content. For instance, CycleGAN has been specifically applied to generate facades that harmonize with traditional architectural styles, as demonstrated by Sun et al. [9]. These technologies assist architects by generating design schemes that incorporate historical features efficiently, reducing the time required for data collection and preliminary design processes. Moreover, they aid in complex data analysis, facilitating the integration of modern functionalities and energy efficiency into traditional forms. This synthesis of generative AI tools supports the preservation of cultural heritage and enhances the utility and sustainability of renovated structures, showcasing a significant leap forward in how architectural design can accommodate and respect historical contexts while embracing modern technology.

In transforming traditional architectural facades into multifunctional commercial styles, this study introduces novel generation and evaluation methods using AI-generated content. The study’s key contributions are threefold. (1) We developed a general framework based on stable diffusion models for the commercial style transfer of historical facades, emphasizing a systematic design process and broad applicability across various building styles. Integrating the low-rank adaptation (LoRA) and ControlNet models enhances the image accuracy and stability, making the approach versatile for architectural modernization. (2) We proposed a combined qualitative and quantitative evaluation method using the contrastive language–image pre-training (CLIP) and Frechet inception distance (FID) scores to comprehensively assess the quality of the generated facades, providing insights for future model optimization. (3) A specialized dataset featuring nighttime shopfront images from the historical districts was created, offering high-quality data support that enhances the image detail and style consistency. This dataset not only meets the study’s experimental needs but also serves as a valuable resource for future research in similar domains.

## 2. Related Works

With the advancement of artificial intelligence technologies, the emergence of deep generative models such as generative adversarial networks (GANs) and stable diffusion models has introduced new possibilities for architectural design and style transfer. These technologies, by learning from vast datasets, can autonomously generate images in designated styles, providing designers with a wealth of inspiration and rapid iteration capabilities for their projects. This research reviews the literature related to GANs and stable diffusion, compares their advantages, and discusses the potential and applications of generative AI in the design of facades for historical Chinese architecture.

### 2.1. Generative Adversarial Networks in Design Transfer

GANs are a type of machine-learning algorithm that generates realistic images through the adversarial competition between two neural networks, further developed by researchers in various directions. GANs have garnered widespread adoption in the realm of image generation, demonstrating remarkable proficiency in crafting high-quality, high-resolution imagery. They hold considerable potential in generating traditional architectural facades and urban redevelopment. In the realm of traditional architectural facades, Yu et al. [13] utilized GANs to generate Chinese traditional architectural facades by training the model on labeled data samples and elements of historical Chinese architecture. Additionally, GANs also play a supportive role in urban redevelopment design. Sun et al. [9] and Ali and Lee [14] have developed tools based on GANs for urban renewal, preserving unique facade designs in urban districts. Khan et al. [15] employed GANs to generate realistic urban mobility networks (MoGAN), thus depicting the entirety of the mobility flows within a city. As such, GANs can significantly enhance the efficiency of facade design during the initial stages of the design process. Employing GAN models can reduce the cultural knowledge requirements for designers, generating a diversity of images with characteristics of traditional architectural facades, thereby facilitating work in the early stages [16].

### 2.2. Research on Diffusion Models in Design Transfer

In comparison to GANs, diffusion models have achieved more favorable outcomes in terms of the image generation quality [17,18]. In the realm of image generation, Ho and Salimans [19] modified diffusion models, introducing the denoising diffusion implicit models (DDIMs). They drastically enhance the speed of sample generation, offering a 10- to 50-fold improvement over denoising diffusion probabilistic models (DDPMs). DDPMs utilize a learned reverse diffusion technique to produce data, whereas DDIMs introduce advancements that significantly expedite this process [20], iteratively denoising to produce clear, high-quality images. The VQ-diffusion model, a text-to-image architecture, is capable of generating high-quality images in both conditioned and unconditioned scenarios, excelling at creating more complex scenes [20]. W. Wang et al. [18] initially tested and validated the efficacy of diffusion models for semantic image synthesis, enhancing the models’ performance and displaying superior visual quality of generated images. While the output of single-step models is not yet competitive with GANs, it significantly surpasses previous likelihood-based single-step models. Future efforts in this area may bridge the sampling speed gap between diffusion models and GANs without compromise [17]. Furthermore, Kim and Ye [21] introduced the diffusion CLIP model, which not only speeds up computations but also achieves near-perfect inversion—a task that presents certain limitations with GANs. As a result, the computational speed gap between diffusion models and GANs is continuously narrowing, while diffusion models have widened the application range compared to GANs.

The stable diffusion model, an evolution of the diffusion model technology from text-to-image, combines large-scale training data and advanced deep-learning techniques to create a more stable and adaptable conditional image generator. A key advantage of the stable diffusion model is its time and energy efficiency [22]. Known for its user-friendly interface, stable diffusion allows users to adjust the interface according to their needs, making it easy for even those inexperienced with AI to generate high-quality images [22]. Jo et al. [23] elucidated an approach that utilizes stable diffusion models to develop alternative architectural design options based on local identity. Users simply input a textual description of the desired image to rapidly generate the corresponding visuals. In terms of usability, diffusion models not only save time but also enable designers to quickly and conveniently experiment with different ideas and concepts. Furthermore, they exhibit superior stability and controllability in terms of style transfer, offering designers a broader creative space and encouraging widespread adoption. Consequently, diffusion models can be more readily embraced by designers and generate higher-quality images.

### 2.3. Lateral Comparison and Limitations

Due to design choices in network structures, target functions, and optimization algorithms, GANs encounter challenges such as mode collapse and instability during training [24]. Training a GAN model requires not only extensive sample data and neural architecture engineering but also specific “tricks” [25]. However, diffusion models also exhibit limitations in terms of their generation stability and content consistency [26]. To be specific, when a new concept is added, diffusion models tend to forget the earliest text-to-image models, thereby reducing their ability to generate high-quality images from the past. However, recent research has shown that the LoRA model holds potential in enhancing the accuracy of stable diffusion-generated images. L. Sun et al. [26] proposed a content-consistent super-resolution approach to improve the training of diffusion models, aiming to enhance the stability of graphic generation. Smith et al. [27] introduced the C-LORA model to address catastrophic past issues in continuous diffusion models. Additionally, Luo et al. [28] proposed that LCM-LoRA can serve as an independent and efficient neural network-based solver module, enabling rapid inference with minimal steps across various fine-tuned SD models and SD LoRA models. Hence, the LoRA model can generate images with stable styles and reduced memory usage, allowing for fine-tuning that further meets designers’ needs in architectural transformations.

However, textual descriptions often fail to generate accurate results and struggle to understand complex text. Thus, additional control conditions are needed alongside textual descriptions to increase the accuracy of image generation. ControlNet is a neural network architecture designed to add spatial conditional control to large pre-trained text-to-image diffusion models, such as stable diffusion [29]. It enables precise control over the generated images using conditions such as edges, depth, and human poses. Zhao et al. [30] proposed Uni-ControlNet, which allows the use of different local and global controls within the same model, further reducing the cost and size of model fine-tuning while enhancing the controllability and combinability of text-to-image transformations. Therefore, we incorporate the ControlNet neural architecture into our model, utilizing the extracted line drawings to control the architectural outlines. This approach aims to reduce the excessive modifications to traditional architectures and explore the efficiency of the model when combining line drawing control conditions with ControlNet [31].

This study aims to explore the technique of commercial style transformation of residential buildings based on the stable diffusion model, proposing the LoRA model for transforming the facades of traditional Chinese residences. Through this model, designers can easily transform the facades of traditional residences into commercially attractive design proposals while preserving the architectural historical characteristics and cultural value. Additionally, we emulate Sun’s data collection method by manually capturing photographs from streets to create the specialized training dataset needed for this model. We also focus more on commercial signage design, incorporating finer architectural details to create a platform that autonomously generates commercialized traditional residences, facilitating both natural heritage conservation and urban cultural tradition preservation [9].

## 3. Method and Material

### 3.1. Methodology

**Figure 1:**
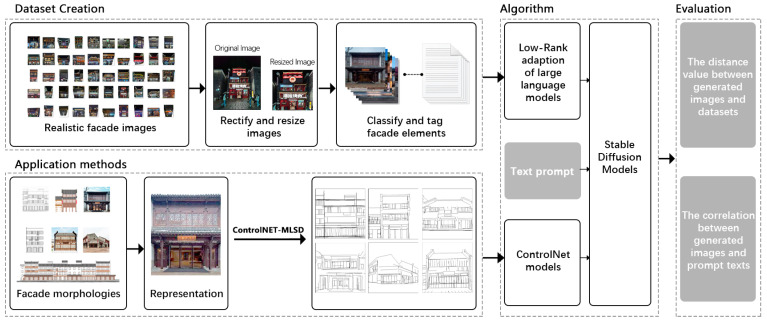
Research flowchart.

This paper describes a methodology utilizing diffusion models for the automated generation of images, which is designed to facilitate the commercial style transformation of traditional residences. This process not only ensures the quality of the transformation but also extends its application to the aesthetic design of various building types. The refined workflow, as depicted in Figure 1, involves several key steps. Initially, commercial facade images of southern residences are collected and curated to establish a high-quality dataset of commercialized traditional residences. This dataset, which is crucial as it guides the process toward the desired outcome, contains vital information on traditional architectural styles. While several open-source facade datasets are available for network training, a proprietary dataset representing the traditional style of a specific district is necessary. Subsequently, each facade image is labeled with textual tags to form the training dataset for the LoRA model, enabling the generation of high-quality, stylized images by the stable diffusion model system. The images generated undergo both qualitative and quantitative assessments. Finally, the ControlNet model is introduced to enhance the accuracy and stability of the outputs, with the workflow efficacy validated against existing cases of traditional residences.

### 3.2. Data Collection and Processing

**Figure 2:**
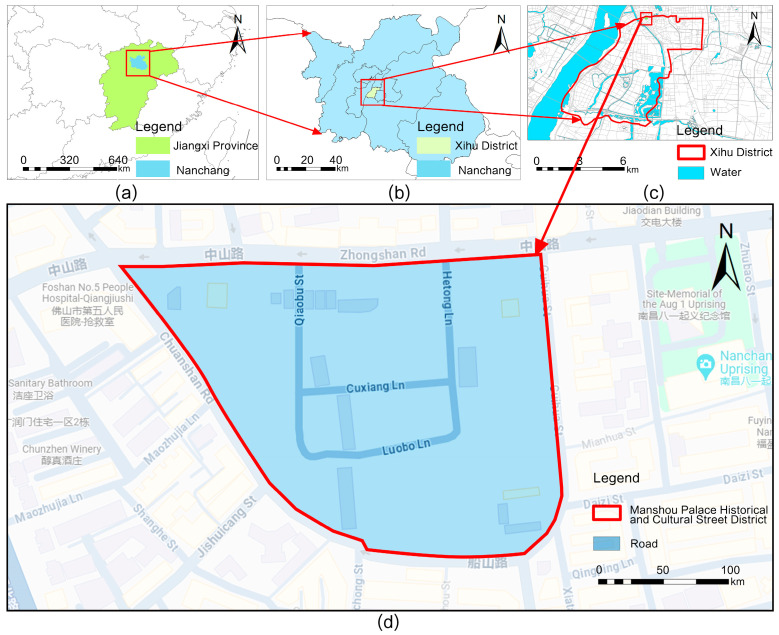
The study area. (**a**) Jiangxi Province, (**b**) Nanchang city, (**c**) Xihu district in Nanchang, and (**d**) the Wanshou Palace Historical and Cultural District.

For this study, Wanshou Palace in Jiangxi Province was selected as the subject. Located in Nanchang City, Jiangxi Province, China (Figure 2), this street is a large, comprehensive urban district. It spans a significant area of 5.46 hectares and preserves valuable wooden residential buildings, representing one of the few urban areas that retain their historical structure and scale.

The commercial sections of the Wanshou Palace Historical and Cultural District feature a variety of traditional architectural facades that have been adapted for commercial use. This district exemplifies how historical architecture can be integrated with modern commercial activities to create a vibrant and historically rich commercial area, making it an outstanding case study for the commercial style transformation of historical architecture. The variety of shop signs within the Wanshou Palace district—differing in form, location, materials, and lighting—poses a design challenge in creating multiple styles quickly to meet client needs while ensuring cohesion within the district street scape.

Compared to traditional design phases, the diffusion model plays a crucial role in this study by learning the commercial style transformation of historical architecture. Additionally, the architectural style complexity of the street makes it an ideal target for testing the proposed method. All the images were manually captured by the authors from the commercial streets, selecting only high-quality shopfront images to ensure the quality and reliability of the facade image dataset. The selection was based on three considerations: (1) the local government’s positive appraisal of the commercial value of the Wanshou Palace Historical and Cultural District, highlighting successful commercial transformations of historical architecture; (2) the lighting and weather conditions, with nighttime images better showcasing the effects of the facade shop signs; and (3) the richness of the architectural details, as the facades of historical architecture typically include complex decorations and details, making high-quality, high-resolution images particularly important.

**Figure 3:**
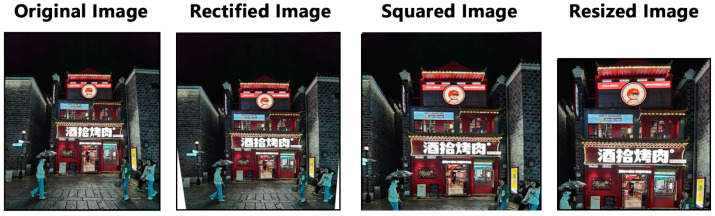
Image processing.

A total of 71 photos were initially taken from the street, with 43 remaining after removing those with incorrect perspectives or severe obstructions by crowds. Each image underwent preprocessing, and textual tags were generated for each using DeepDanbooru. Figure 3 illustrates the preprocessing steps taken for each image, including correction, squarization, and uniform adjustment to a resolution of 512 × 512 pixels.

**Figure 4:**
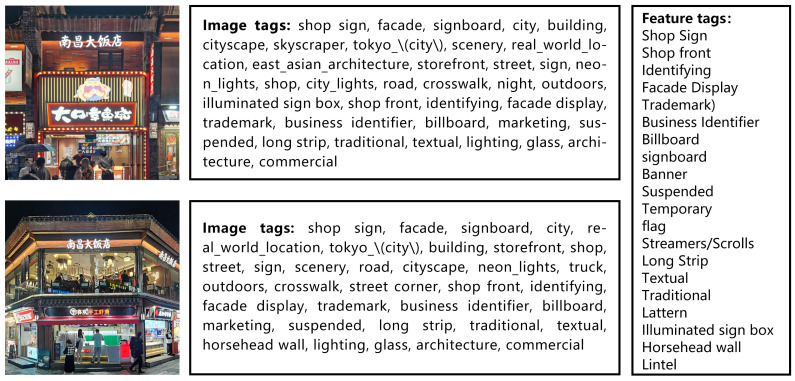
Image prompt word labels.

Based on the generated textual tags, specific textual labels were applied to each image according to the characteristic elements of the commercial architectural facades. These specific textual labels included the location of the shop signs (entrance lintels, shop fronts along the street), the structure of the shop signs (signboards, awnings) and the arrangement of the shop signs (parallel or vertical). Figure 4 shows examples of traditional architectural shop sign facades paired with their corresponding textual labels. This rigorous tagging and preprocessing lay the foundation for the subsequent training of the LoRA model and the generation of stylized commercial facade images.

### 3.3. Model Training and LoRA Model Generation

The LoRA model achieves efficient adaptation to specific tasks by incorporating low-rank matrices within critical layers of the model. For this study, 43 images of nighttime traditional architectural shopfronts were used to define a specific image domain. The objective was for the model to learn and generate new images with similar styles and elements.

We selected v1-5-pruned.safetensorsas [32] as the base and adapted self-attention layers or other critical layers of the model to accommodate the visual characteristics of the nighttime historical architecture.

Suppose W is the weight matrix of a layer in the pretrained model. We introduce two low-rank matrices A and B to modify this weight. The adjusted weights $\tilde{W}$ can be represented as:

(1) $$\tilde{W}=W+AB^{T}.$$

The adjustment and training process involves modifying the original weights by the low-rank matrices to form new weight matrices W, while keeping most parameters of the pretrained model unchanged, only training these low-rank matrices [33]. The 43 images and their annotations are used to train these low-rank matrices, adapting the model for the task of generating images of traditional architectural shopfronts at night. These matrices are specifically designed to capture the visual effects of architectural elements and lighting conditions at night.

The generative network architecture of the LoRA model uses two downsampling convolution layers with a stride of 2, followed by five residual blocks to process image features, and then two upsampling convolution layers with a stride of 1/2 to generate the target images. All the non-residual block convolution layers are followed by instance normalization to accelerate the training and enhance the generation quality [33].

During training, the LoRA model aims to minimize the difference between the generated images and the actual nighttime shopfront images. This is achieved through a combination of reconstruction loss and regularization loss of the low-rank matrices:

The reconstruction loss ($L_{recon}$) measures the difference between the generated images and the target nighttime images. If x is the input image, y is the target image, and G is the generator (including weight adjustments), then the reconstruction loss can be expressed as:

(2) $$L_{recon}=∥G\left(x\right)-y{∥}_{2}^{2},$$

The regularization loss ($L_{reg}$) is intended to prevent overfitting and maintain the model generalization capability, and a regularization loss is applied to the low-rank matrices A and B:

(3) $$L_{reg}=\lambda \left(∥A{∥}_{F}^{2}+∥B{∥}_{F}^{2}\right),$$

Here, ‖.‖F denotes the Frobenius norm, and λ is the weight of the regularization term, which is used to balance the impact of both losses.

The total loss $L_{total}$ for the LoRA model is a combination of these two losses:

(4) $$L_{total}=L_{recon}+L_{reg},$$

Using these two primary loss functions, during training, the values of the low-rank matrices A and B are adjusted by minimizing the total loss $L_{total}$. This involves a loss based on the parameter updates of the low-rank matrices A and B and an evaluation loss for the quality of generated images, which might include pixel-level or feature-level losses to ensure that the visual quality of the generated images matches the original nighttime images.

## 4. Results

### 4.1. Commercial Style LoRA Model and Generated Outcomes

**Figure 5:**
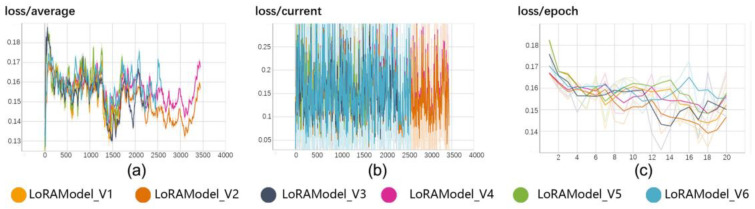
LoRA models’ training loss. (**a**) shows the average loss over all iterations, indicating the overall trend of the model’s learning progress. (**b**) displays the current loss for each iteration, providing a detailed view of the immediate changes in loss during training. (**c**) illustrates the loss per epoch, showing how the loss evolves after each complete pass through the training dataset.

In our research endeavor, we successfully trained six LoRA models on a single NVIDIA GeForce RTX 3090 graphics card, utilizing a uniform dataset of traditional architectural shopfront facade images in conjunction with the v1-5-pruned.safetensors base model. Table 1 presents a detailed account of the training parameters employed for these six LoRA models. Throughout the training phase, we meticulously optimized and adjusted the models by manipulating critical parameters such as the batch size, repeat factor, and learning rate, with the objective of enhancing their performance capabilities. During this process, we continuously recorded the loss values of the models in real time, which served as a fundamental basis for evaluating the models’ learning efficacy and guiding strategic adjustments to the training regimen. Figure 5 graphically illustrates the loss values observed across the six LoRA models.

**Figure 6:**
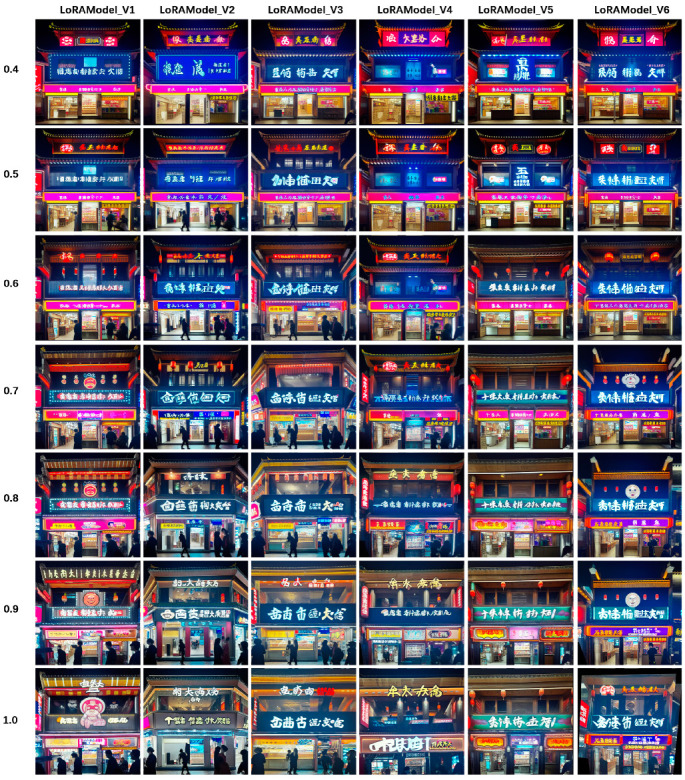
The images generated by 6 LoRA models were compared under different weights.

We conducted comprehensive and detailed comparative testing of commercial-style facade generation utilizing the six trained LoRA models to ensure the selection of the model with optimal performance. Figure 6 presents a comparative illustration of the traditional architectural facades generated from text prompts using the LoRA models within stable diffusion, with the horizontal axis representing different LoRA models and the vertical axis indicating the weight values of the LoRA models. We tested the performance of the LoRA models across various weight settings ranging from 0.4 to 1. The comparative figure reveals that different LoRA models exhibit varying degrees of responsiveness to the prompts at different weight values, with Model 2 demonstrating superior results at weight values between 0.7 and 0.9. Specifically, the facades generated under these conditions effectively and accurately trigger the labels within the prompts, resulting in clear and vibrant overall imagery. Taking into account all the factors, we selected the outstanding model for fine-tuning the larger model within stable diffusion, which generates traditional architectural shopfront images from textual prompts. Compared to authentic images, it is evident that the generated shopfront facades have accurately captured the nuances essential to the transformation of traditional architectural commercial styles. Rather than merely replicating existing facade data, our model demonstrates a comprehensive learning and understanding of the form, placement, and lighting colors of shopfronts. These results underscore the potential of our model to provide robust technical support for the renovation and revitalization of urban historic districts.

### 4.2. Quantitative Analysis

In this study, a series of experiments were designed to evaluate the performance of different versions of LoRA models under various weight configurations. The experiments employed two key performance metrics: the CLIP score and the FID score. The CLIP score assesses the model performance on language–image alignment tasks, while the FID score measures the quality of the generated images. By utilizing these metrics, we can obtain a comprehensive understanding of the model performance. Each weight setting of every LoRA model generated 200 images under randomly assigned seeds, from which 50 images were randomly selected to calculate both the FID and CLIP scores.

#### 4.2.1. Analysis of FID Scores

During the training of the LoRA models, which are used to tune a larger model for image generation tasks—either by integration with an image generation model or by adjusting the model to handle image-related inputs and outputs—the oscillatory nature of the loss function makes it unreliable to directly assess the model performance through loss curves. For this reason, the FID is employed as an evaluation metric [34]. The FID score operates by comparing the distribution of generated images to that of real images within a feature space. This feature space is extracted using a pre-trained deep-learning model, typically the Inception v3 model [35]. The FID calculates the mean and covariance of the features of both generated and real images, and then computes the Frechet distance (also known as Wasserstein-2 distance) between these distributions. A lower FID score indicates a closer distribution of generated images to real images, implying the higher quality of the generated images and thus reflecting superior model performance. The FID has become an essential tool for assessing performance in image generation tasks, especially where direct evaluation of the model performance using loss curves is challenging. The stability of the FID and its consistency with human visual perception make it a reliable metric for evaluating LoRA models.

Research indicates that the range of the FID scores can vary depending on the dataset and model architecture. For instance, GAN models generating handwritten digits on the MNIST dataset exhibit FID scores ranging from 78.0 to 299.0 [36], while models trained on higher-resolution natural image datasets show FID scores between 6.9 and 27 [37]. Additionally, studies on high-resolution pre-labeled datasets have reported FID scores ranging from 22.6 to 104.7 [38], where Sun reported FID ranges from 85 to 145.9. Consequently, for the facade dataset used in this study, by applying the LoRA model across different facade datasets, a benchmark range of 140–190 [9] provides a reference standard, aiding in the understanding and evaluation of the LoRA model performance on specific datasets.

#### 4.2.2. Analysis of FID Scores across Different LoRA Model Versions

**Figure 7:**
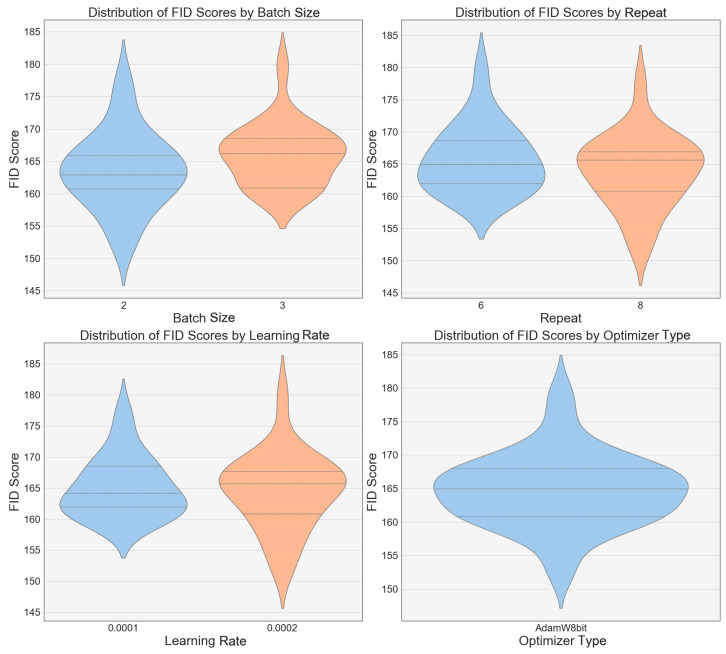
FID score analysis for different versions of the LoRA models.

The FID scores for several LoRA models are displayed, indicating that the image quality has improved with the model updates (Figure 7). Significant variations in the distribution of the FID scores are noted under different batch sizes, learning rates, and types of optimizers. Particularly, under a configuration with a batch size of 2, a learning rate of 0.0002, and using the AdamW8bit optimizer, the models produced the highest quality images with the lowest FID scores, notably Model 2, as previously mentioned.

**Figure 8:**
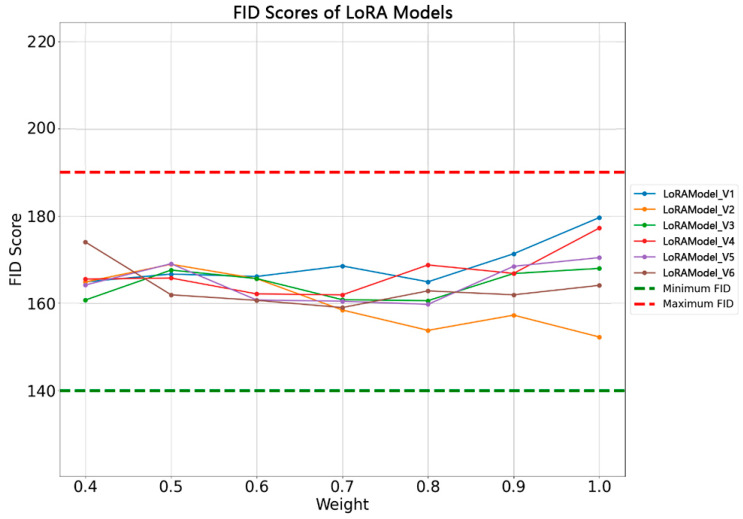
FID scores for differently weighted LoRA models.

The range of FID scores for all the models is within a certain threshold (indicated by a green dashed line for the minimum FID and a red dashed line for the maximum FID). This suggests that all the models perform within an acceptable range, yet no single model is optimal across all the weight values. The variation in the FID scores under different weight configurations indicates that the model performance fluctuates with changes in the weight (Figure 8). Most models appear to perform better at weights approximately between 0.7 and 0.8, where almost all the versions of the models achieve their lowest FID scores, although the specific optimal weight may vary by model. As the weights increase, most models show a fluctuating trend in the FID scores, which may indicate that different weight settings inconsistently affect each model, potentially involving specific characteristics of the model structure or the training process.

#### 4.2.3. Analysis of CLIP Scores across Different LoRA Model Versions

**Figure 9:**
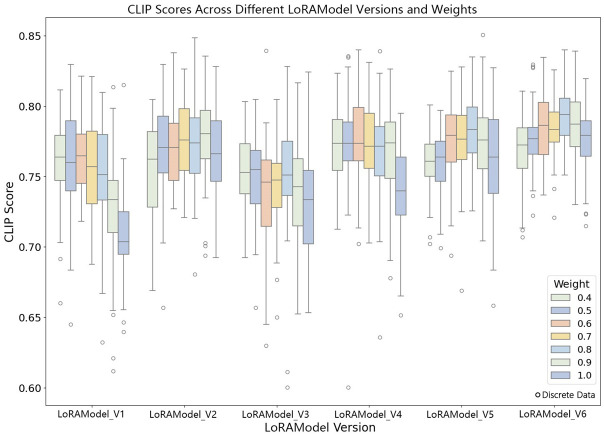
CLIP scores for different LoRA model versions and weights.

The experimental results show significant fluctuations in the CLIP scores across different versions of the LoRA models as the models are iteratively updated (Figure 9). Each version was tested under varying weight parameters, and the median CLIP scores for each version exhibited various degrees of increase or decrease as the weights changed from 0.4 to 1.0, suggesting that weight adjustments significantly impact the model performance. For example, LoRAModel_V3 performs best at a weight of 0.7, while LoRAModel_V2 reaches its peak performance at a weight of 1.0. This may be because increasing the weight to a certain level better captures the mapping relationships between language and images. Overall, the median CLIP score at a weight of 0.7 is usually higher than other weight configurations, indicating that this weight setting may be more suitable for language–image alignment tasks within the current model architecture.

**Figure 10:**
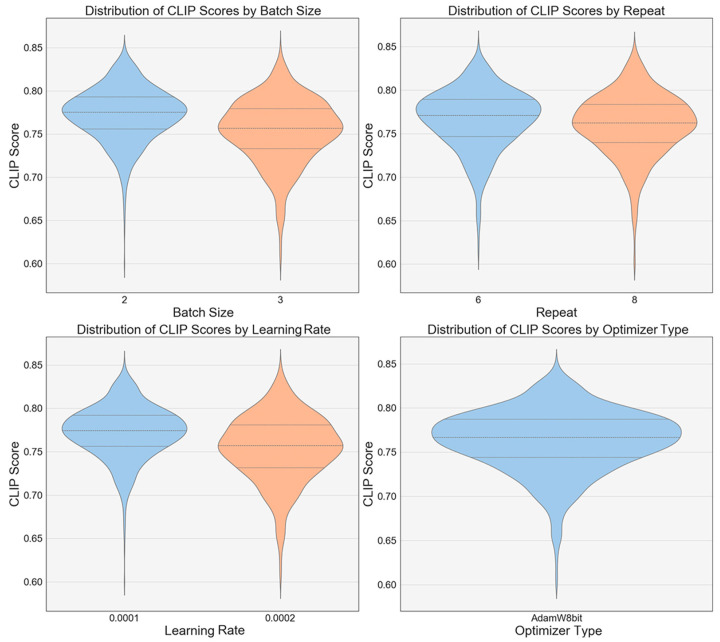
CLIP score analysis of different versions of the LoRA models.

Further experimental analysis examined the impact of hyperparameters such as the batch size, repeat count, learning rate, and type of optimizer on the CLIP scores through a violin plot analysis (Figure 10). The results show that a batch size of 2 yields a higher median and more stable CLIP scores compared to configurations with a batch size of 3, implying that smaller batch sizes contribute to enhanced model performance. Increasing the number of repetitions appears to have a positive effect on the model stability, particularly at 8 repetitions, where the coefficient of variation of the CLIP scores is minimal, resulting in a tighter score distribution. Regarding the learning rates, a setting of 0.0001 results in a more compact distribution of the CLIP scores, indicating that this rate is favorable for improving the model performance. In terms of the optimizer choice, the AdamW optimizer generally achieves higher CLIP scores than the Lion optimizer across most scenarios.

### 4.3. Qualitative Analysis

While the FID and CLIP scores provide a preliminary assessment of the image quality and text–image alignment, they are subject to potential errors. To mitigate these errors and enhance the precision and consistency of the results, we also conducted a qualitative analysis by visually comparing the generated images with actual scenes to assess their realism and consistency. According to Borji’s [39] analysis, this method is the most direct and widely used evaluation approach. Given that this study primarily focuses on whether models can transition traditional historical facades into a commercial style, our qualitative analysis concentrated on architects’ preferences and the visual effects of the model-generated images. To minimize the impact of personal biases and subjectivity, we invited 10 architectural researchers to rate the generated images across three dimensions: “image quality”, “alignment with traditional architectural style”, and “commercial effect of the facade”. The ratings were quantified on a scale from 1 to 10, with the intervals defined as follows: [0, 2) “very low”, [2, 4) “low”, [4, 6) “medium”, [6, 8) “high”, [8,10] “very high”, to quantify the degree of fit for each criterion. The evaluation process was divided into two stages: initially, participants rated each model’s images under each weight setting; subsequently, they assessed images generated by the same model under different weights. By aggregating and averaging all the researchers’ scores, we obtained a quantified qualitative analysis.

**Figure 11:**
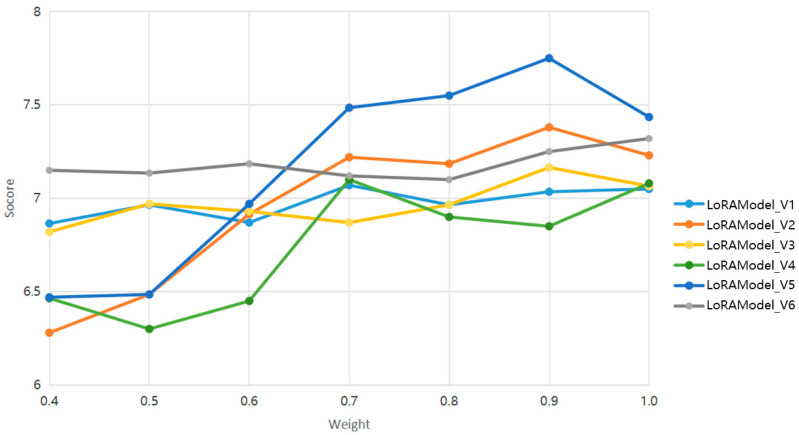
Qualitative analysis weights of the different LoRA models as scored by experts.

As shown in Figure 11, a weight analysis of the LoRA model series was conducted to evaluate its performance in the commercial transformation application of historical architecture. The results indicated that within the weight range of 0.7 to 0.9, LoRAModel_v2 and LoRAModel_v5 scored significantly higher than the other models, demonstrating better adaptability, suggesting that these two models are more suited to meeting the specific needs of traditional architectural transformation. Meanwhile, LoRAModel_v6 and LoRAModel_v3 showed a consistent scoring trend across the entire range of weights, with LoRAModel_v6 slightly outperforming in all the scenarios. The results of this qualitative analysis not only affirm the LoRA models capability to effectively convert traditional residential facades into commercial style facades but also offer novel technical pathways for the commercial transformation of historical architecture. Additionally, these findings provide a basis for subsequent model selection and optimization, ensuring that the chosen models achieve the best style transfer effects under specific transformation requirements.

### 4.4. Applications in Different Scenarios

**Figure 12:**
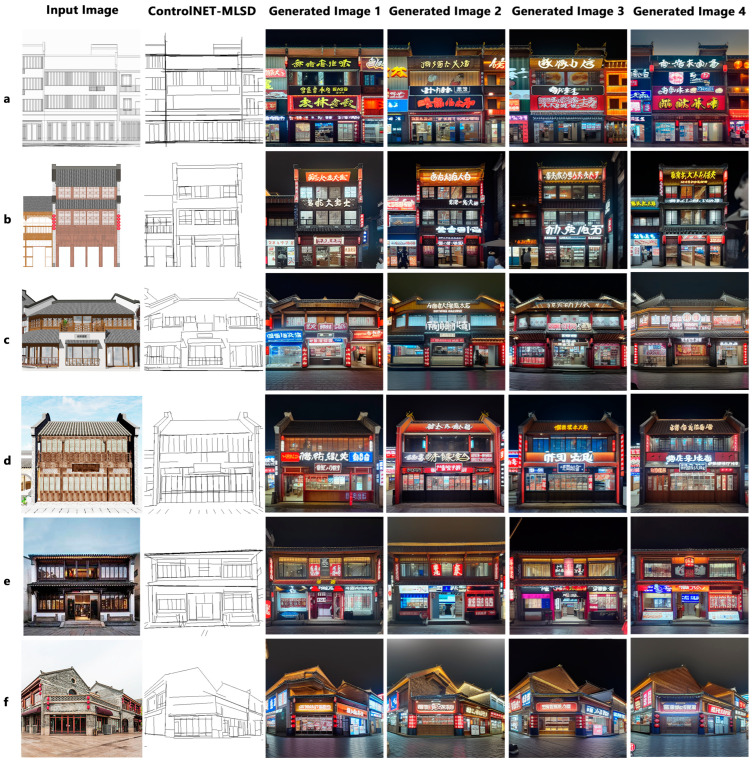
The generated images for commercial style transfer in different application scenarios based on prompts and ControlNet: (**a**) style conversion of wireframes, (**b**) style conversion of simulated elevations, (**c**,**d**) style transformation of renderings, and (**e**,**f**) style conversion of live photos.

**Figure 13:**
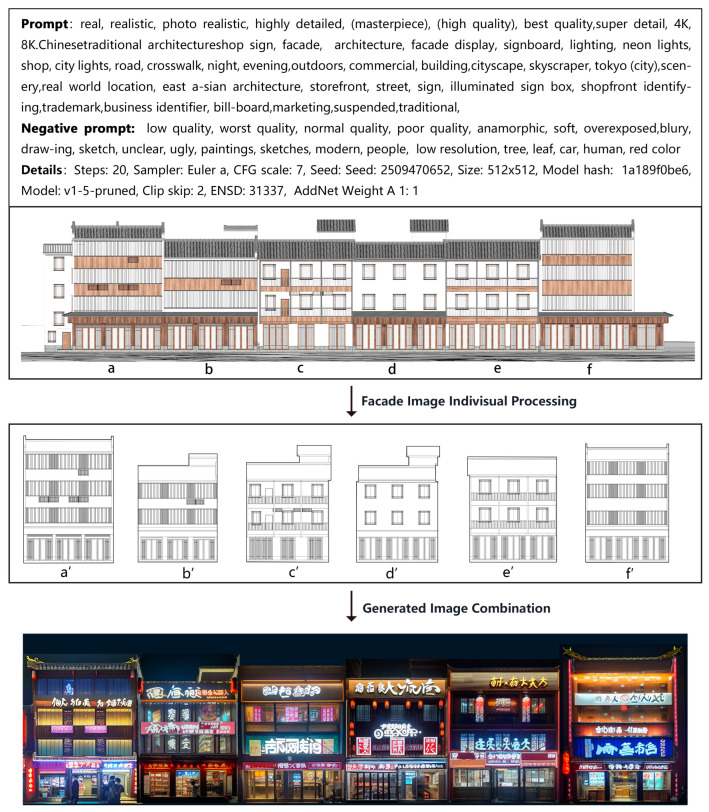
The overall facade style of the commercial street was rearranged based on above method. (**a**–**f**) are different individual building facades combined to form a street facade, while (**a’**–**f’**) are the corresponding line drawings of the individual building facades.

We endeavor to apply this innovative methodology to commercial style transformation projects of traditional architectures, offering more efficient and personalized solutions for their commercial renewal. By leveraging the sketches drawn by architects and on-site photographs provided, we input these constraint information into ControlNet as control conditions during the style transfer of traditional architectures. The utilization of these conditions significantly enhances the precision and relevance of the images generated by our model. Figure 12 showcases the facade renewal generation results based on the prompt descriptions and existing images. The results indicate that when the preprocessed outcomes from ControlNet’s MLSD model are employed as control conditions, the generated storefront facades exhibit controllable changes compared to their pre-transformation states. These alterations extend beyond the mere facade of the buildings, influencing even the overall appearance of the entire block, aligning more closely with our design objectives. These controllable variations encompass the design and replacement of storefront styles, positions, lighting fixtures, and other components, along with a notable improvement in the overall harmony, as shown in Figure 13. These meticulous modifications are automatically accomplished by our model, drastically reducing the design and revision time while ensuring the quality of the design schemes.

In this study, the demonstrated image synthesis effects achieve remarkable levels in terms of the realism, style consistency, and detail presentation. Regarding realism, the synthetic images visually approximate real scenes, featuring rich colors and textures, and the effects of lighting and shadows are vividly life-like, showcasing the model’s accurate capability in capturing lighting effects and material textures. In terms of the style consistency, each generated image maintains a high degree of uniformity in style, reflecting the model’s accurate interpretation and the reproduction capability of the commercial style inputs, such as universally recognizable commercial logos, prominent signs, and lighting elements, which are typical expressions of commercial architectural style. In detail handling, the model cleverly integrates commercial elements while preserving the original architectural structures, such as well-designed signs and window advertisements, which enrich the visual effect and enhance the commercial atmosphere, demonstrating the model’s profound understanding of interpreting and reproducing architectural functionality. Additionally, although all the images adhere to the same commercial style, they exhibit a rich diversity in color coordination, sign design, and brand display, reflecting the traits of a high-quality synthesis model—that is, while being faithful to a set style, it still manages to create personalized and diverse images.

## 5. Discussion

This study aims to present a systematic method for automatically generating the facades of traditional buildings in a commercial style and to explore the application effectiveness of this method in the design of traditional building shopfronts, thereby verifying its feasibility. A central consideration of this study is whether this method can enhance the efficiency and outcomes of traditional architectural preservation processes. However, the method requires further in-depth research and refinement.

Firstly, to enhance the diversity and realism of the learning samples, it is necessary to incorporate more case studies. Training the LoRA model with nighttime shopfront photos from specific locations, such as the Wanshou Palace Historical and Cultural District in Nanchang in China, may show superior performance for specific applications, like recognizing or generating nighttime facade images from specific locations. However, choosing nighttime photos from specific locations might limit the diversity of the dataset. This lack of diversity could impact the model’s generalizability, potentially underperforming when processing a broader range of images. Additionally, since the photos are taken at night, the model might overly adapt to nighttime lighting conditions, color distributions, and shadow effects, leading to poorer performance under daytime or other lighting conditions. In some cases, it might be necessary to expand the dataset to include photos from different times of the day or similar scenes from other locations to enhance the diversity.

In terms of the experimental results, the FID scores reported in this paper are relatively high compared to other studies, possibly due to the dataset primarily containing specific nighttime scenes. While the FID scores are a useful metric for assessing the quality of natural images, the inception model may not effectively capture the key features of images from specific domains, such as cartoons, abstract art, or images with a specific style, leading to distorted FID scores. Nevertheless, the FID scores should not be the sole metric for evaluating generative models. This study integrates the results of qualitative and quantitative evaluations to select the best-performing LoRA model for application in research. In the preliminary phase of architectural design, utilizing digital technology to aid design not only improves the efficiency of architects but also promotes efficient communication with clients. Thus, the evaluation methods proposed in this study offer valuable reference points for another research.

In modernizing historical architecture, particularly during commercial transformations, preserving its cultural and historical value is crucial. The LoRA model, through deep learning, can understand and reproduce the style and details of historical architecture, helping designers find a balance in blending the old with the new. This ensures that the renovation plans not only meet modern needs but also respect and preserve the original spirit and cultural significance of the architecture.

## 6. Conclusions

This paper presents a method leveraging diffusion models to convert traditional residences into commercial styles. We initially curated a dataset of commercial facades from southern residences, crucial for transforming traditional architectural styles. Annotating image elements to train the LoRA model enabled the diffusion models to generate high-quality, stylized images. The image quality was validated through qualitative and quantitative assessments, and the incorporation of the ControlNet model enhanced the accuracy and stability. The method’s application in existing traditional residences demonstrated its effectiveness and potential in architectural design.

The experimental results confirm this workflow’s reliability in preserving traditional historical architecture. We efficiently generated high-quality images of traditional buildings with commercial style transformations, providing architects with creative ideas for modern transformations. This study challenges traditional design constraints and explores new possibilities for historical architecture conservation. The key contributions include: (1) proposing a design process for transitioning traditional residences into commercial styles, allowing architects to quickly generate user-expected designs; (2) establishing combined qualitative and quantitative evaluation methods for assessing commercial style images of traditional buildings, offering crucial feedback for generative model optimization; and (3) providing a pre-trained LoRA model for commercial style transformation, enhancing the design efficiency and quality.

However, the method may be influenced by architects’ experience and perception, potentially limiting comprehensive keyword provision during prompt input for image generation. In historical architecture renewal, architects should select the appropriate control network model based on specific application needs to ensure consistent high-quality outputs. This study focused on traditional residences’ commercial transformation. Future research could refine the process for different stages, enhance the workflow convenience and efficiency, and expand the dataset by creating diverse traditional architectural facade datasets. This would broaden the stable diffusion model’s application, produce diverse results, and consider the operational space of traditional cultural districts. Additionally, combining 2D image generation with 3D modeling to create visualization models of historical commercial street transformations merits further exploration.

## Figures and Tables

**Table 1:** LoRA models’ training parameters.

Color	LoRA Model	Batch Size	Epoch	Repeat	Learning Rate	Optimizer Type
** 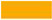 **	LoRAModel_V1	3	20	6	0.0002	AdamW8bit
** 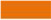 **	LoRAModel_V2	2	20	8	0.0002	AdamW8bit
** 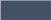 **	LoRAModel_V3	3	20	8	0.0002	AdamW8bit
** 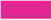 **	LoRAModel_V4	2	20	8	0.0001	AdamW8bit
** 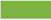 **	LoRAModel_V5	3	20	6	0.0001	AdamW8bit
** 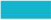 **	LoRAModel_V6	2	20	6	0.0001	AdamW8bit

## Data Availability

Data will be made available on request.

## References

[1] Alexandrakis G., Manasakis C., Kampanis N.A. (2019). Economic and Societal Impacts on Cultural Heritage Sites, Resulting from Natural Effects and Climate Change. Heritage.

[2] Ng W.-K., Hsu F.-T., Chao C.-F., Chen C.-L. (2023). Sustainable Competitive Advantage of Cultural Heritage Sites: Three Destinations in East Asia. Sustainability.

[3] Bullen P.A., Love P.E.D. (2010). The Rhetoric of Adaptive Reuse or Reality of Demolition: Views from the Field. Cities.

[4] Saarinen J. (2010). Local Tourism Awareness: Community Views in Katutura and King Nehale Conservancy, Namibia. Dev. S. Afr..

[5] Franklin A. (2004). Tourism as an Ordering: Towards a New Ontology of Tourism. Tour. Stud..

[6] Tang P., Wang X., Shi X. (2019). Generative Design Method of the Facade of Traditional Architecture and Settlement Based on Knowledge Discovery and Digital Generation: A Case Study of Gunanjie Street in China. Int. J. Archit. Herit..

[7] Xie S., Gu K., Zhang X. (2020). Urban Conservation in China in an International Context: Retrospect and Prospects. Habitat Int..

[8] Charter A. The Athens Charter for the Restoration of Historic Monuments. Proceedings of the First International Congress of Architects and Technicians of Historic Monuments.

[9] Sun C., Zhou Y., Han Y. (2022). Automatic Generation of Architecture Facade for Historical Urban Renovation Using Generative Adversarial Network. Build. Environ..

[10] Stoica I.S. (2020). Imaginative Communities: Admired Cities, Regions and Countries, RobertGoversReputo Press, Antwerp, Belgium, 2018. 158 pp. $17.99 (Paper). Governance.

[11] Goodfellow I., Pouget-Abadie J., Mirza M., Xu B., Warde-Farley D., Ozair S., Courville A., Bengio Y. (2020). Generative Adversarial Networks. Commun. ACM.

[12] Zhu J.-Y., Park T., Isola P., Efros A.A. (2020). Unpaired Image-to-Image Translation Using Cycle-Consistent Adversarial Networks. arXiv.

[13] Yu Q., Malaeb J., Ma W. (2020). Architectural Facade Recognition and Generation through Generative Adversarial Networks. Proceedings of the 2020 International Conference on Big Data & Artificial Intelligence & Software Engineering (ICBASE).

[14] Ali A.K., Lee O.J. (2023). Facade Style Mixing Using Artificial Intelligence for Urban Infill. Architecture.

[15] Khan A., Lee C.-H., Huang P.Y., Clark B.K. (2023). Leveraging Generative Adversarial Networks to Create Realistic Scanning Transmission Electron Microscopy Images. NPJ Comput. Mater..

[16] Haji S., Yamaji K., Takagi T., Takahashi S., Hayase Y., Ebihara Y., Ito H., Sakai Y., Furukawa T. (2023). Façade Design Support System with Control of Image Generation Using GAN. IIAI Lett. Inform. Interdiscip. Res..

[17] Nichol A., Dhariwal P. Improved Denoising Diffusion Probabilistic Models. Proceedings of the 38th International Conference on Machine Learning.

[18] Wang W., Bao J., Zhou W., Chen D., Chen D., Yuan L., Li H. (2022). Semantic Image Synthesis via Diffusion Models. arXiv.

[19] Ho J., Salimans T. (2022). Classifier-Free Diffusion Guidance. arXiv.

[20] Gu S., Chen D., Bao J., Wen F., Zhang B., Chen D., Yuan L., Guo B. (2022). Vector Quantized Diffusion Model for Text-to-Image Synthesis. Proceedings of the 2022 IEEE/CVF Conference on Computer Vision and Pattern Recognition (CVPR).

[21] Kim G., Ye J.C. Diffusionclip: Text-Guided Image Manipulation Using Diffusion Models. Proceedings of the 2022 International Conference on Learning Representations.

[22] Yıldırım E. Text to Image Artificial Intelligence in a Basic Design Studio: Spatialization from Novel. Proceedings of the 4th International Scientific Research and Innovation Congress.

[23] Jo H., Lee J.-K., Lee Y.-C., Choo S. (2024). Generative Artificial Intelligence and Building Design: Early Photorealistic Render Visualization of Façades Using Local Identity-Trained Models. J. Comput. Des. Eng..

[24] Saxena D., Cao J. (2022). Generative Adversarial Networks (GANs): Challenges, Solutions, and Future Directions. ACM Comput. Surv..

[25] Kurach K., Lučić M., Zhai X., Michalski M., Gelly S. A Large-Scale Study on Regularization and Normalization in GANs. Proceedings of the International Conference on Machine Learning, PMLR.

[26] Sun L., Wu R., Zhang Z., Yong H., Zhang L. (2023). Improving the Stability of Diffusion Models for Content Consistent Super-Resolution. arXiv.

[27] Smith J.S., Hsu Y.-C., Zhang L., Hua T., Kira Z., Shen Y., Jin H. (2023). Continual Diffusion: Continual Customization of Text-to-Image Diffusion with C-LoRA. arXiv.

[28] Luo S., Tan Y., Patil S., Gu D., von Platen P., Passos A., Huang L., Li J., Zhao H. (2023). LCM-LoRA: A Universal Stable-Diffusion Acceleration Module. arXiv.

[29] Zhang L., Rao A., Agrawala M. (2023). Adding Conditional Control to Text-to-Image Diffusion Models. Proceedings of the 2023 IEEE/CVF International Conference on Computer Vision (ICCV).

[30] Zhao S., Chen D., Chen Y.-C., Bao J., Hao S., Yuan L., Wong K.-Y.K. Uni-ControlNet: All-in-One Control to Text-to-Image Diffusion Models. Proceedings of the Advances in Neural Information Processing Systems 36.

[31] Wang T., Zhang T., Zhang B., Ouyang H., Chen D., Chen Q., Wen F. (2022). Pre-training Is All You Need for Image-to-Image Translation. arXiv.

[32] Rombach R., Blattmann A., Lorenz D., Esser P., Ommer B. (2022). High-Resolution Image Synthesis with Latent Diffusion Models. Proceedings of the 2022 IEEE/CVF Conference on Computer Vision and Pattern Recognition (CVPR).

[33] Hu E.J., Shen Y., Wallis P., Allen-Zhu Z., Li Y., Wang S., Wang L., Chen W. (2021). LoRA: Low-Rank Adaptation of Large Language Models. arXiv.

[34] Bynagari N.B. (2019). GANs Trained by a Two Time-Scale Update Rule Converge to a Local Nash Equilibrium. Asian J. Appl. Sci. Eng..

[35] Szegedy C., Vanhoucke V., Ioffe S., Shlens J., Wojna Z. (2016). Rethinking the Inception Architecture for Computer Vision. Proceedings of the 2016 IEEE Conference on Computer Vision and Pattern Recognition (CVPR).

[36] Lucic M., Kurach K., Michalski M., Gelly S., Bousquet O. Are Gans Created Equal? A Large-Scale Study. Proceedings of the Advances in Neural Information Processing Systems 31.

[37] Brock A., Donahue J., Simonyan K. (2019). Large Scale GAN Training for High Fidelity Natural Image Synthesis. arXiv.

[38] Park T., Liu M.-Y., Wang T.-C., Zhu J.-Y. Semantic Image Synthesis with Spatially-Adaptive Normalization. Proceedings of the IEEE/CVF Conference on Computer Vision and Pattern Recognition.

[39] Borji A. (2019). Pros and Cons of GAN Evaluation Measures. Comput. Vis. Image Underst..

